# The Importance of Vaccination in the Context of the COVID-19 Pandemic: A Brief Update Regarding the Use of Vaccines

**DOI:** 10.3390/vaccines10040591

**Published:** 2022-04-12

**Authors:** Bruna Aparecida Souza Machado, Katharine Valéria Saraiva Hodel, Larissa Moraes dos Santos Fonseca, Vinícius Couto Pires, Luis Alberto Brêda Mascarenhas, Leone Peter Correia da Silva Andrade, Marcelo Albano Moret, Roberto Badaró

**Affiliations:** 1 SENAI Institute of Innovation (ISI) in Health Advanced Systems (CIMATEC ISI SAS), University Center SENAI/CIMATEC, Salvador 41650-010, Brazil; katharine.hodel@fieb.org.br (K.V.S.H.); larissa.fonseca@fieb.org.br (L.M.d.S.F.); vinicius.pires@fbter.org.br (V.C.P.); breda@fieb.org.br (L.A.B.M.); leone@fieb.org.br (L.P.C.d.S.A.); moret@fieb.org.br (M.A.M.); badaro@fieb.org.br (R.B.); 2 UNEB, Universidade do Estado da Bahia, Salvador 41150-000, Brazil

**Keywords:** COVID-19, vaccines, SARS-CoV-2, COV, pandemic

## Abstract

The COVID-19 pandemic has led the world to undertake the largest vaccination campaign in human history. In record time, unprecedented scientific and governmental efforts have resulted in the acquisition of immunizers utilizing different technologies (nucleotide acids, viral vectors, inactivated and protein-based vaccines). Currently, 33 vaccines have already been approved by regulatory agencies in different countries, and more than 10 billion doses have been administered worldwide. Despite the undeniable impact of vaccination on the control of the pandemic, the recurrent emergence of new variants of interest has raised new challenges. The recent viral mutations precede new outbreaks that rapidly spread at global proportions. In addition, reducing protective efficacy rates have been observed among the main authorized vaccines. Besides these issues, several other crucial issues for the appropriate combatting of the pandemic remain uncertain or under investigation. Particularly noteworthy issues include the use of vaccine-boosting strategies to increase protection; concerns related to the long-term safety of vaccines, child immunization reliability and uncommon adverse events; the persistence of the virus in society; and the transition from a pandemic to an endemic state. In this review, we describe the updated scenario regarding SARS-CoV-2 variants and COVID-19 vaccines. In addition, we outline current discussions covering COVID-19 vaccine safety and efficacy, and the future pandemic perspectives.

## 1. Introduction

The rapid spread and contagion of severe acute respiratory syndrome coronavirus-2 (SARS-CoV-2), the etiologic agent of coronavirus disease 2019 (COVID-19), raised concern among the public and health authorities worldwide. Shortly after the first case was reported in Wuhan (China), the World Health Organization (WHO) defined COVID-19 as a pandemic [1,2]. Since the pandemic began, one of the main effective and feasible ways to contain the spread of the SARS-CoV-2 has been through vaccination [3]. Therefore, with unprecedented efforts, multiple vaccine candidates have been identified to prevent SARS-CoV-2 infection in a noticeable period [4]. The “race” to develop COVID-19 vaccines has been characterized primarily by the use of different technology platforms, such as nucleic acid (mRNA and DNA) [5,6,7,8], viral vector (replicating and non-replicating) [9,10,11], inactivated virus [12], and protein-based technologies [13]. Currently, more than 180 vaccines are under clinical development worldwide, while 33 vaccines have already been approved by regulatory agencies in different countries [14]. As a result of the immense efforts made by governmental and independent organizations, as well as the pharmaceutical and biotech industries, in just over 1 year since the first COVID-19 vaccine was approved for emergency use [15], more than 10 billion doses have been administered worldwide, representing the largest vaccination program in human history [16].

Despite this motivating context, the current COVID-19 pandemic scenario is concerning due to the emergence of new SARS-CoV-2 variants. In general, coronaviruses are large RNA viruses, with the largest among them being characterized as non-segmented and single-stranded positive-sense RNA (+ssRNA) [17]. SARS-CoV-2 has a 29.9 kb genome capable of encoding four structural proteins (spike, envelope, membrane, and nucleocapsid) and sixteen non-structural proteins (nsp1-16) [18]. Among them, the spike (S) protein of SARS-CoV-2 plays a critical role in the virus, as it is responsible for binding to receptors (angiotensin-converting enzyme 2, or ACE2) on the host cell and determining host tropism [19]. The S protein is composed of two functional subunits named S1 and S2, which are responsible for binding to the receptor on the host cell and for fusing the viral membrane to that of the host cell, respectively [20,21]. 

Even though SARS-CoV-2 features molecular tools for nucleotide revision during viral replication, such as the RNA-dependent RNA polymerase complex (RdRP), this process is still error-prone [22]. Coupled with this, the high prevalence of COVID-19 worldwide has led to the rapid emergence of viral variants that exhibit characteristics differing from the original virus [23]. Within this context, modifications to the S protein have resulted in increased pathogenicity and higher viral transmission capacity, as well as negatively impacting vaccine efficacy and the performance of available diagnostic kits, which has attracted the attention of health authorities [24,25,26]. The WHO and other public health institutions have classified SARS-CoV-2 variants into two main categories: variants of concern (VOCs) and variants of interest (VOIs). In general, VOCs are the SARS-CoV-2 lineages that have been confirmed to be capable of increased transmissibility, virulence, vaccine resistance, decreased immunity acquired from previous infections, and the ability to evade diagnostic detection. VOIs have alterations that may decrease the action of neutralizing antibodies generated from vaccines or previous infections, decrease the effectiveness of available therapies, and have an impact on the transmissibility, severity, or diagnosis of the disease [27].

In November 2021, a new VOC named Omicron (B.1.1.529) was identified in Botswana (South Africa), and it rapidly escalated to epidemiological proportions [28]. The Omicron variant has been responsible for the increase in the number of COVID-19 cases in distinct parts of the world in early 2022 [29], with more than 1 million new cases being reported in a single day in the United States alone, with the Omicron variant dominating in most of them [30]. The dominance of infections caused by Omicron may be associated with its greater transmissibility, which is up to 3.2 times higher when compared to other VOCs [31,32]. This property has been associated primarily with Omicron’s ability to evade the immune system in individuals previously immunized with the primary vaccination scheme or who have had previous infection through other SARS-CoV-2 lineages [33]. In addition, Omicron presents important particularities that have resulted in new directions related to COVID-19 dynamics, such as a shorter incubation period (2 to 3 days) when compared to the original strain (5 to 7 days) [34]; a lower affinity for lung cells, decreasing the severity of respiratory symptoms [35]; as well as a higher reinfection rate, indicating important immune-system escape mechanisms [36]. Given these characteristics, it is believed that the COVID-19 situation could take a drastic turn due to transmission of the Omicron variant, as well as other upcoming VOCs with even greater transmission properties, increasing the importance of community immunization [37,38].

Therefore, the unpredictability of how the COVID-19 pandemic will progress in the face of the emergence of new variants has caused health authorities and vaccine developers to search for strategies to mitigate the impacts caused by mutant lineages. The expansion of the eligible population for vaccination, such as the inclusion of children between 5 and 11 years old, and the recommendation for booster doses after primary vaccination are strategies that have fostered discussions related to the efficacy, safety, and the role of vaccines developed at the beginning of the pandemic. Therefore, the aim of this study was to evaluate the expectations related to the COVID-19 pandemic regarding the use of vaccines. 

## 2. Overview of COVID-19 Vaccines and Variants

One of the biggest challenges for vaccination, especially when it comes to airborne viruses—such as flu viruses, for example—is the emergence of new variants caused by mutations in the virus genome. Mutations occur naturally during the replication of any RNA virus due to the instability of the RNA molecules [23]. In the case of COVID-19, even before it was considered a pandemic, data related to the genomic surveillance of SARS-CoV-2 were available as a useful tool for investigating outbreaks and tracking evolution and possible new waves [39,40]. As a result, more than 25 billion sequences of SARS-CoV-2 have been performed worldwide, and it is estimated that at least two mutations in the viral genome occur per month [40,41]. These additional mutations often result in distinct immune-evasion mechanisms and lead to the appearance of different variants and lineages [42]. Currently, there are 21 variants of SARS-CoV-2; among them, variants Alpha a (B.1.1.7), Beta (B.1.351), Gamma (P.1), Delta (B.1.617.2), and Omicron (B.1.1.529) are considered as VOCs, while Lambda (C.37) and Mu (B.1.621) are considered as VOIs (Table 1). It is emphasized that, in addition to these two categories, there are also variants under monitoring (VUMs), which are those strains that have genetic mutations that may pose a risk in the future, but for which phenotypic and epidemiological changes are currently unclear [43].

Because one of their characteristics is lower susceptibility to vaccines and other therapeutic alternatives, VOCs have been intensively monitored [44]. Modifications found in the structure of the S protein of SARS-CoV-2 have been attributed to the greater ability of the virus to escape the action of neutralizing antibodies [43,45]. Important examples of mutational mechanisms that lead to increased antigenic properties of protein S are the amino acid substitutions that alter the protein epitope, increase receptor-binding avidity, and lead to changes in glycosylation, deletion or insertion of residues, and allosteric structural effects [46]. These factors strongly contribute to the increased mortality and morbidity of SARS-CoV-2 [46,47,48], where the transmissibility can be up to 74% higher when compared to the original strain [49,50].

Since the SARS-CoV-2 S protein is the main target of COVID-19 vaccines, the mutations in this protein are of great concern, especially those which have the corresponding sequences of reference strain Wuhan-Hu-1—that is, no antigens based on different variants are used [51,52]. Thus, the appearance of variants with modifications in the SARS-CoV-2 S protein structure raises questions about the effectiveness of the vaccines available to the population; antibodies derived from the original strain (Wuhan-Hu-1) may have only a partial neutralizing effect against these viruses [53]. The vaccines BNT162b2 (brand name Comirnaty), mRNA-1273 (brand name Spikevax), CoronaVac, BBIBP-CorV, AZD-1222 (brand name Vaxzevria or Covishield), and Ad26.COV2-S (brand name Janssen COVID-19 Vaccine) are the most widely used around the world for COVID-19 prophylaxis, since all of them use the S protein as the main activator of the immune system [54]. Therefore, different studies have been published or are being conducted to analyze the efficacy or effectiveness of each of these vaccines against SARS-CoV-2 variants of concern (Table 2).

**Figure 1:**
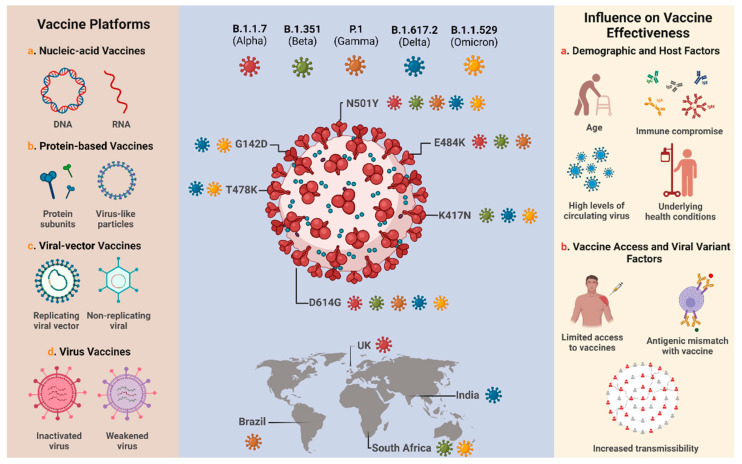
Overview of the major technology platforms used for COVID-19 vaccine development, the SARS-CoV-2 variants of concern and their respective spike protein mutations, and the factors that may influence the effectiveness of available vaccines. Adapted from Tregoning et al. [44] and Mistry et al. [94]. Created with BioRender.com (accessed on 17 February 2022).

Analysis of several studies shows that the efficiency or effectiveness of vaccines against SARS-CoV-2 variants depends on many factors, including the sample size, demographic factors, host factors, the type of vaccine, the number of doses, a heterologous or homologous booster vaccination scheme, and the time after primary vaccination is completed (Figure 1) [44,84,85]. Different authors demonstrated that the application of a booster dose after a certain period is able to increase the humoral immune response against SARS-CoV-2, resulting in increased efficacy or effectiveness of vaccines against the VOC [86,87,88,89,90]. Bruxvoort et al. [91] found data that reinforced the need for booster dose administration, since primary immunization with mRNA-1273 shows limited protection against the Delta, Alpha, Gamma, and Mu variants. Andrews et al. [78] reported that administration of the booster dose with BNT162b2 after primary immunization of ChAdOx1, mRNA-1273, or BNT162b2 was able to significantly increase protection against Omicron. Other authors reported that the homologous booster regimen of BNT162b2, mRNA-1273, CoronaVac and BBIBP-CorV vaccines and the heterologous booster with Ad26.COV2.S associated with mRNA-1273 vaccines showed similar performance against Omicron [92,93]. 

Data provided by the CDC show that the number of cases and deaths caused by COVID-19 in the United States is higher among unvaccinated individuals when compared to individuals with a full primary vaccination scheme and/or those who have already received a booster dose, regardless of the vaccine administered (BNT162b2, mRNA-1273, or Ad26.COV2-S) [95]. Among those vaccinated, although the three vaccines showed similar efficacy in reducing COVID-19 infection in the period evaluated (April 2021 to February 2022), it was observed that the number of deaths among individuals vaccinated with Ad26.COV2-S was higher when compared to the mRNA vaccines (BNT162b2 and mRNA-1273) [95]. In early January 2022, during the wave caused by the Omicron variant, the highest incidence per 100,000 population occurred, where the rates reached 5.44, 2.34, and 1.79 for the Ad26.COV2-S, BNT162b2, and mRNA-1273 vaccines, respectively, after the primary vaccination scheme [95]. However, it is important to note that the mortality rate among those who received the Ad26.COV2-S vaccine was lower compared to unvaccinated individuals, where the rate was ~9 times higher. Although it has already been reported that the Ad26.COV2-S vaccine can elicit a stable humoral and cellular response over time, data reported by the CDC suggest that the immune response induced by mRNA vaccines may be more effective in reducing mortality rates [83,96]. The greater efficacy observed in mRNA vaccines may be a reflection of their advantages as a technological platform; unlike adenovirus (DNA)-based vaccines, once administered, RNA molecules do not need to cross the nuclear membrane or transcription to start the protein-synthesis process [97]. Hence, these data encouraged discussions about the immune protection of the Ad26.COV2-S vaccine, particularly with a view to the possible implementation of the intervals required for the application of new booster doses.

Because of this, since the second half of 2021, health authorities around the world have been recommending booster doses for different vaccines to contain the spread of SARS-CoV-2 and consequently control the pandemic in terms of the number of hospitalizations and deaths [98,99,100,101]. The study by Andrews et al. [102] conducted in England demonstrated that the administration of a booster dose with mRNA vaccines (BNT162b2 or mRNA-1273) compared to the primary immunization showed an efficiency between 94 and 97% in reducing symptomatic cases of the disease, while against hospitalization or death, the value found ranged between 97% and 99%. Studies conducted in Israel have also shown the same trend, which strengthens the importance of this new vaccination strategy [103,104,105]. Furthermore, studies involving computer modeling have shown that the administration of the booster dose will be able to decrease the effective reproduction number (R_0_), while authors have pointed out that the predicted increase in antibody titers induced by the booster dose provides important protection from infection with SARS-CoV-2 variants [106,107,108].

Currently, the application of a new booster dose (usually the fourth dose) has been recommended in different countries due to the emergence of the Omicron variant, which has a greater transmission capacity than the other variants and, consequently, has been associated with an increase in the number of reinfection cases [109,110,111]. Initially, the administration of this new dose was directed towards priority groups, such as healthcare workers, immunocompromised individuals, and the elderly; however, new groups are expected to be included soon [112,113]. A preliminary study released by the Israeli Ministry of Health in adults aged >60 years demonstrated that a fourth dose of the BNT162b2 vaccine was able to increase immune protection up to two-fold against SARS-CoV-2 infection and up to three-fold against severe disease when compared to individuals who received only the third dose [114]. The expectation that new VOCs will emerge over time exposes one of the main challenges associated with developing COVID-19 vaccines, that of using products capable of inducing a robust and/or long-lasting immune response against different variants [115,116]. 

In early November, a new COVID-19 sublineage named BA.2 was first reported [117] (2 months after variation BA.1, which rapidly became dominant due to immune-escape mechanisms [118,119]). In a public statement, WHO defined it as an Omicron sublineage, classifying it as a variant of concern. According to the organization, the amino acids differences in structural proteins have possibly conferred a growth advantage when compared to other Omicron sublineages (BA.1, BA. 1.1.), but not greater severity [120]. In later March, the US Centers for Disease Control and Prevention (CDC) reported that BA.2 was responsible for 55% (50.8–59.1%—95% PI) COVID-19 cases [121], followed by BA. 1.1 (40.4%, 36.4–44.5%—95% PI). The rapid spread of BA.2 has raised discussions about reinfection and vaccine efficacy. Although BA.2’s ability to evade neutralizing antibodies is unclear, authors have demonstrated evidence that the increasing frequency of BA.2 is probably related to increased transmissibility rather than to enhanced immunologic escape [122]. On the other hand, initial data from population-level reinfection studies suggest that infection with BA.1 provides strong protection against reinfection with BA.2 [123]. Recent studies have demonstrated that mRNA vaccines (BNT162b2 and mRNA-1273) provide similar protection against BA.1 (46.6% (95% CI: 33.4–57.2%) and BA.2 51.7% (95% CI: 43.2–58.9%) in the first three months, and this declines to about 10% after 4–6 months. These findings show that protection against BA.2 did not seem to wane any faster than protection against BA.1. Furthermore, in both cases, a second dose was able to recuperate immune protection levels [124]. Therefore, until now, recent data supported the need for a vaccine targeting the Omicron variant.

Within this context, in January 2022 the companies Pfizer and BioNTech, developers of the BNT162b2 vaccine, started a clinical trial to evaluate the safety, immunogenicity, and tolerability of an Omicron-based vaccine candidate. To this end, the study will be conducted with healthy adults between the ages of 18 and 55 who may be allocated into three distinct cohorts with different dose regimens of the vaccine candidate [125]. Similarly, the pharmaceutical company Moderna (developer of the mRNA-1273 vaccine) is expected to start a clinical trial for the analysis of a new vaccine candidate against the Omicron variant in the first half of 2022 [126]. In addition, different institutes have supported the idea of investing in the development of a pan-coronavirus vaccine capable of protecting against several coronaviruses, including the different strains of SARS-CoV-2 [126,127]. 

Importantly, the development of Omicron-specific vaccines should not completely rule out the use of previously approved vaccines made available to the population, as robust data are still needed to elucidate the induction of the immune response after the first booster dose and its role in controlling infection or disease progression by this variant. Furthermore, one cannot exclude the possible “selective pressure” exerted by vaccines and even by monoclonal antibody therapy in targeting the S protein, since this may have influenced the appearance of new variants with mutations in this region, thus conferring escape mechanisms [128]. Therefore, new therapeutic targets must be considered for the development of new vaccines. 

## 3. Safety of COVID-19 Vaccines

Due to the pandemic nature of COVID-19 and the various impacts generated in the global health, social and economic sectors, the vaccines against SARS-CoV-2 infection were made available in record time [129]. This was also because many scientists, manufacturers, and research institutions were already developing innovative technology platforms for new vaccines, which were eventually adapted for COVID-19 prevention. However, since no coronavirus vaccine had been licensed and approved for use in humans previously, the rapid development associated with the limited follow-up time post-vaccination and lack of information about long-term side effects of the vaccines aroused great public concern about the safety profile of the available vaccines [130]. It is important to note that as mass vaccination progresses, more post-vaccination adverse events are reported [131]. This demonstrates that vaccine safety information from ongoing clinical trials and surveillance data is important not only for building public confidence, but also for making evidence-based health-policy decisions [132]. The safety of vaccines is evaluated through adverse event monitoring in randomized controlled trials and safety post-licensure surveillance data after immunization campaigns [133]. Determining the safety profile of a vaccine is a critical step at the global level and is monitored by the WHO along with manufacturers, health officials, and national regulatory agencies [5,6,134], since they are drugs administered in healthy populations. According to the WHO, all available vaccines, including the COVID-19 vaccines, have been rigorously assessed for safety for diverse groups of people, according to age, sex, ethnicity, and medical conditions.

For the mRNA vaccines, the most commonly reported adverse events are local reactions at the injection site, such as pain, redness, and swelling, and systemic reactions, such as headache, myalgia, arthralgia, and chills [135]. In clinical studies evaluating the mRNA-1273 and BNT162b2 vaccines, the frequency and severity of these adverse events were higher after the administration of the second dose. When it comes to adenoviral vector vaccines, in the case of the AZD 1222 vaccine, pain, fever, chills, muscle ache, headache, and malaise were the most common adverse reactions. Regarding serious adverse events, seven have been associated with the AZD-122 vaccine, including transverse myelitis [136]. Overall, inactivated virus vaccines such as CoronaVac, BBIBP-CorV, and COVXIN have a good safety profile, with few grade 3 adverse reactions. In the elderly population, studies with vaccines of distinct technologies, such as AZD 1222 (modified adenovirus) and NVX-CoV2373 (protein adjuvant), for example, showed a good antibody response and low reactogenicity events after administration, with a higher incidence and severity of adverse events observed in younger subjects [137]. 

Another rare manifestation after vaccination, but which has been reported in different studies, is multisystem inflammatory syndrome (MIS) [138]. MIS has still poorly understood pathophysiology, however, it is believed to occur due to an exaggerated immune response against SARS-CoV-2 infection due to persistently high levels of IgG and activation of CD8+ T cells [139,140]. Considering the adult population, MIS may result from a delayed and dysregulated immune response and is characterized by the onset of symptoms such as fever, elevated inflammatory markers, as well as multiple-organ involvement (especially of the heart, stomach, and intestines) [141,142]. Different case studies have reported the onset of MIS in adults after immunization with the vaccine based on mRNA [143], inactivated virus [144], and adenovirus [145]. However, a crucial question was demonstrated by Belay et al. [146], in which the report of MIS after vaccination in adult patients was also associated with prior SARS-CoV-2 infection. These data demonstrate the need for further studies to elucidate the real association between MIS caused purely by vaccination or whether there is a direct relationship with previous viral infection.

It is interesting to highlight that most of the side effects that people experience after COVID-19 vaccination can be attributed to the “nocebo” effect. Nocebo refers to the non-pharmacological adverse effects reported after exposure to a placebo substance, which are usually motivated by the individual’s expectation that, after exposure to a vaccine, drug, or other medical intervention, some disagreeable event will occur [147,148]. Haas et al. [149] evaluated the frequency of adverse events in the placebo arm in 12 clinical studies of COVID-19 vaccines (mRNA-1273, CoV2 preS dTM, NVX-CoV2373, AZD-1222, BNT162b2, BNT162b1, and SCB-2019) at different phases of clinical development, and the results found demonstrate that while adverse events were mostly reported in the arms receiving the experimental vaccines, subjects receiving the placebo reported a significant frequency of adverse events. These results highlight the importance of critically evaluating the safety of experimental vaccines, especially when some minority groups are known to be resistant to COVID-19 vaccination [150,151]. 

When it comes to serious adverse events, particular concern has emerged related to the safety of COVID-19 during pregnancy due to reported cases of thrombosis and thrombocytopenia syndrome after vaccination with AZD 1222 in early 2021 [152]. Despite the devastating consequences of COVID-19 infection in pregnant women and the availability of vaccine safety and efficacy data in different populations, data related to vaccine safety in pregnant women are still limited, since most of the ongoing clinical trials do not include pregnant women [153]. However, preclinical and toxicological COVID-19 vaccine studies have found no safety concerns with no adverse effect on female reproduction, fertility, fetal or embryonal, or postnatal development, or miscarriage [153,154,155]. For mRNA vaccines, surveillance data demonstrated that vaccine-related adverse events in pregnant women were similar to those in non-pregnant women, with pain in the local area of the injection, fatigue, headache, and myalgia being the most frequent local and systemic reactions after vaccination [153]. Regarding the safety of COVID-19 vaccines for the fetus or breastfeeding infant, various expert panels suggest that mRNA-based and adenovirus vector vaccines do not possess any significant risk [156,157].

### COVID-19 Vaccines and Serious Adverse Events: Myocarditis and Pericarditis

Currently, the main safety concerns for COVID-19 vaccines are related to mRNA vaccines, with the emergence of cases of myocarditis and pericarditis. Myocarditis is the inflammation of the heart muscle, while pericarditis is the inflammation of the outer lining of the heart. In both cases, the immune system causes inflammation in response to an infection or some other factor. Both can occur during infections, including SARS-CoV-2 infection. In the case of inflammation caused by mRNA vaccines, one of the first articles involving the evaluation of the incidence of myocarditis after vaccination with Pfizer’s vaccine (BNT162b2 or Comirnaty) was published in October 2021 in the New England Journal of Medicine. The study was conducted with patients in a large Israeli healthcare system who had received at least one dose of the vaccine. The authors reported an estimated incidence of myocarditis of 2.13 cases per 100,000 people; the highest incidence was among male patients between the ages of 16 and 29. Most cases of myocarditis were mild or moderate in severity [158]. According to the CDC, myocarditis is a rare and serious adverse event that has been associated with mRNA-based COVID-19 vaccines, in this case BNT162b2. The reporting rates of vaccine-associated myocarditis appear to be highest among males aged 12–29 years. As of 31 December 2021, myocarditis among children aged 5–11 years is classified as rare, where 11 Vaccine Adverse Event Reporting System (VAERS)-verified reports were received after the administration of approximately eight million doses of vaccine, and in an active vaccine safety surveillance system, no confirmed reports of myocarditis were observed during the 1–21 days or 1–42 days after 333,000 doses of vaccine were administered to children of the same age. Two deaths following the BNT162b2 vaccine were reported in children with multiple chronic medical conditions, where, in the initial review, no data were found to suggest a causal association between death and vaccination. 

It is important to compare the cases among vaccinated and infected ones. The incidence of COVID-19-associated cardiac injury or myocarditis can be 100 times higher than COVID-19 mRNA-vaccine-related myocarditis [159,160]. Another relevant observation is that cases of myocarditis and pericarditis have been reported mainly for mRNA vaccines [161]; in Brazil, no cases have been reported related to inactivated virus-based vaccines [162]. The possible mechanisms underlying heart injury side-effects in specific groups were brightly reviewed and hypothesized by Heymans and Cooper [159], where, according to the authors, mRNA vaccines might trigger immune hyper immunity in a minority population that is genetically susceptible to developing acute myocarditis after viral injury [163]. In summary, they indicated three potential mechanisms: hormonal differences (the sex-specific distinction can be explained by hormone-related factors); mRNA immune reactivity (genetic variants in HLA genes); and antibodies to SARS-CoV-2 spike glycoproteins cross-reacting with myocardial contractile proteins (genotypes in desmosomal, cytoskeletal or sarcomeric protein).

## 4. Immunization in Children

Although COVID-19 morbidity and mortality are significantly lower in children than in adults, the risk of severe COVID-19 is not negligible even among healthy individuals. A recent report published by United Nations Children’s Fund (UNICEF), with age- and sex-disaggregated data from 104 countries, shows that children and adolescents under 20 years of age account for 18% of the reported COVID-19 cases and 33% of the population [164]. In the United States, recent data from the American Academy of Pediatrics show that by 21 February 2022, 12.3 million pediatric COVID-19 cases had been reported since the onset of the pandemic, which represents 15.7% of all confirmed cases in the country [121,165]. In the case of upper-middle-income countries, such as Brazil, the pediatric public has been neglected in terms of pandemic surveillance. Until February 2022, there was no federal public policy promoting early diagnosis at a federal level. This, associated with the absence of a testing strategy and the screening of suspected cases, makes it difficult to obtain data regarding the incidence of COVID-19 in children and adolescents and, consequently, the preventive and prophylactic measures taken by the public [166,167].

In general, most of the cases of the disease in children and adolescents are mild compared to those in adults. However, according to the American Academy of Pediatrics, the incidence of severe disease is 1% and the rate of death is 1/10,000 [165]. Several cases of multisystemic inflammatory syndrome have also been reported in this population group [168]. Moreover, even after mild cases of COVID-19, children can also present long-term side effects (known as long COVID-19) [121,169]. It is important to highlight that, according to the CDC, the peak of COVID-19 hospitalization in children and adolescents occurred during the Delta- and Omicron-predominant periods, on 11 September 2021 and 8 January 2022, respectively [170]. These data highlight how COVID-19 can represent a serious threat to the lives and health of children and adolescents. Therefore, with the development and availability of new vaccines around the world, there is great concern about the vaccination of this population group.

Among adolescents (12–17 years of age), some health and regulatory agencies have granted emergency use authorization for different COVID-19 vaccines: BNT162b2, mRNA 1273, CoronaVac, and Covaxin [171]. Canada was the first country to approve the vaccine for preventing SARS-CoV-2 infection in May 2021 for this population [172]. When it comes to children (5–11 years of age), vaccination around the world is not yet widely available. Countries such as the United States, Brazil, Canada, China, Israel, Italy, Chile, Australia, and Japan have already authorized or started the vaccination of children between 5 and 11 years of age (Table 3) [173]. The vaccines BNT162b2 and CoronaVac are the main vaccines used for the immunization of children. Except for China, Hong Kong, Chile, Ecuador, Indonesia, Cambodia, and Brazil, which have authorized the use of CoronaVac for children, the other countries that have included the vaccination of children in their immunization plans only use BNT162b2 [174].

The decision to immunize children involves a complex risk–benefit analysis. Therefore, it is important to note that the approval of pediatric vaccination for COVID-19 was neither immediate nor widely accepted. Different opinions have generated discussions from the divergent views among regulatory agencies and government officials. For example, in Brazil, after technical analysis, the Anvisa authorized the pediatric version of the BNT162b2 vaccine on 16 December 2021. However, the Ministry of Health of the country did not consider the decision, claiming that the endorsement of the vigilance agency was not enough, initiating an unprecedented medium of public consultation on the subject. This decision was fostered by the resistance of some parents to immunizing their children. According to preliminary data from Oswaldo Cruz Foundation (Fiocruz), this resistance is related to misinformation about vaccination [175]. After further analysis, vaccination was then approved for children, but not made mandatory, in January 2022 [176].

Recently, the American Academy of Pediatrics reported that by mid-January 2022, about 8 million US children had received at least one dose of the COVID-19 vaccine, representing 28% of children ages 5–11. Considering both doses, 5.3 million U.S. children are fully vaccinated, representing 19% of children ages 5 to 11 [177]. In China, more than 84 million children between 3 and 11 have been vaccinated against COVID-19 with CoronaVac, and more than 49 million of them have received a booster dose [178]. It is important to highlight that the dose level of the BNT162b2 vaccine for children is different from that recommended for adolescents and adults, at 10 and 30 µg, respectively. Another difference is reported in the number of doses per vial, where for adolescents and adults there are six (dosage: 0.3 mL), while for children there are 10 (dosage: 0.2 mL). However, the recommended interval between the first and second dose is the same, regardless of age, and varies according to the regulatory agency of each country (3 weeks, according to the CDC, and 8 weeks according to the Brazilian Health Regulatory Agency—Anvisa) [179]. For CoronaVac, the recommendations made by Anvisa remain the same as for adults, where the two doses should be applied 28 days (about 4 weeks) apart, and the dosage does not change, remaining at 0.5 mL. The application of CoronaVac is not recommended for immunocompromised children.

**Table 3:** Vaccines approved for children immunization worldwide.

Country	Vaccine	Date of Approval for Children (5–11 Years of Age)	Agency	Beginning of Child Immunization	References
United States	BNT162b2 (Pfizer and BioNTech)	29 October 2021	FDA	2 November 2022	[180]
Brazil	BNT162b2 (Pfizer and BioNTech)	16 December 2021	Anvisa	14 January 2022	[181,182]
	CoronaVac (Sinovac)	20 January 2022			
Canada	BNT162b2 (Pfizer and BioNTech)	19 November 2021	Health Canada	23 November 2021	[183]
China	Covilo (Sinopharm Beijing)	8 June 2021 (For 3–11 years of age)	NMPA	25 October 2021	[184,185]
	Vero Cells (Sinopharm Wuhan)				
	CoronaVac (Sinovac)	August 2021			
Israel	BNT162b2 (Pfizer and BioNTech)	14 November 2021	Health Ministry	22 November 2021	[186]
Australia	BNT162b2 (Pfizer and BioNTech)	5 December 2021	TGA	10 January 2022	[187]
Italy	BNT162b2 (Pfizer and BioNTech)	1 December 2021	AIFA	16 December 2021	[188]
Japan	BNT162b2 (Pfizer and BioNTech)	31 January 2022	Japan’s Health Ministry	Forecast to February end or early March 2022	[189]
United Kingdom	BNT162b2 (Pfizer and BioNTech)	22 December 2021 (Vulnerable children) 16 February 2022 (All children)	MHRA	End of January 2022 (vulnerable children) Forecast to mid-April 2022 (All children)	[190]
Chile	CoronaVac (Sinovac)	6 September 2021 (For 6–12 years of age)	ISP	6 December 2021	[191]

AIFA—Agenzia Italiana del Farmaco; Anvisa—Brazilian Health Regulatory Agency; FDA—US Food and Drug Administration; ISP—Instituto de Salud Publica; MHRA—UK Medicines and Healthcare products Regulatory Agency; NMPA—National Medical Products Administration; TGA—Therapeutic Goods Administration.

The safety related to vaccination among the pediatric public is one of the main concerns of health authorities, as well as of public opinion. However, studies have shown that most of the adverse events related to COVID-19 vaccines have been mild to moderate, and resolve within 24 h [192]. The incidence of these adverse reactions has been associated with the dose of the vaccine. For the BNT162b2 vaccine, the most common adverse event in the clinical studies with children and adolescents was pain at the injection site. In addition, headaches, fatigue, and fever were also frequently reported. Most adverse events were not serious, and deaths were not reported [193,194]. Notably, a case report following 13 patients with solid tumors also showed that mild to moderate injection-site pain was the most frequent adverse event (found in six patients) [195]. However, the administration of the BNT162b2 vaccine in the pediatric public has raised different questions regarding the appearance of myocarditis and pericarditis [192]. 

When it comes to CoronaVac, the vaccine has already been applied in members of the public from 2 to 17 years old. According to Anvisa, and considering the international scenario, 86% of the adverse events recorded in this age group are of the non-severe type [196]. In addition, according to the Brazilian Agency, serious adverse events observed after the administration of more than 85 million doses of CoronaVac in Brazil in the public over 18 years old in Brazil are considered rare. Moreover, based on information given by one of the developers of the immunizer (the Butantan Institute), no cases of myocarditis, pericarditis, or thrombosis have been identified in China, where CoronaVac has been administered to the pediatric public for months [162].

As in adults, there is concern about the emergence of MIS cases after COVID-19 vaccination in children, which is also known as Pediatric Inflammatory Multisystem Syndrome Temporally Associated with SARS-CoV-2 (PIMS-TS) [139]. MIS among children is also considered to be a dysregulated immune response, where increased levels of neutralizing antibodies have been reported, leading to a condition of hyper inflammation [197]. Studies conducted in the United States have shown that whether immunization schedules are complete or not, COVID-19 vaccination is safe, whereas cases of MIS in this population after vaccination are rare [198,199]. Specifically, Zambrano et al. [199] demonstrated that immunization with two doses of an mRNA vaccine was able to prevent MIS in the 12- and 18-year-old population. Studies considering the vaccine-eligible population between the ages of 5 and 11 are still scarce in the literature, exposing, as for other public, the need for more incisive research on MSI.

Regarding efficacy, the BNT162b2 vaccine’s efficiency in preventing symptomatic infection in children is similar to that found for adolescent and adult populations, at >90%, even at a lower dose level. A study is being conducted with about 4650 children, where participants received either the experimental vaccine or a placebo [193,200]. As for CoronaVac, phase III clinical trials are ongoing with 14,000 children aged 6 months to 17 years from five different countries (Chile, Malaysia, Philippines, Turkey, and South Africa); however, preliminary results have shown that the immunizer is well tolerated among the pediatric audience. According to data released by Anvisa, an effectiveness study conducted in Chile demonstrated that CoronaVac has an effectiveness of >90% fourteen days after the second dose in participants between 6 and 16 years old, taking hospitalization into account. However, most of the data related to the mentioned studies are still blinded and should be presented [201]. It is also noteworthy that the phase I and II trials conducted in China demonstrated the immunogenicity of the immunizer in the population between 3 and 17 years after two doses, whereas in phase I, 71 individuals participated, and 28 days after vaccination, 100% of them showed antibodies. In phase II, the evaluation with 479 children and adolescents showed that in the group that received a dose of 1.5 µg, 96% of the participants showed antibodies, and in the group that received 3 µg, this number was 100% [202]. These data demonstrate that most COVID-19 vaccines provide good effectiveness and safety; therefore, double-dose vaccinations have been recommended by health authorities.

**Figure 2:**
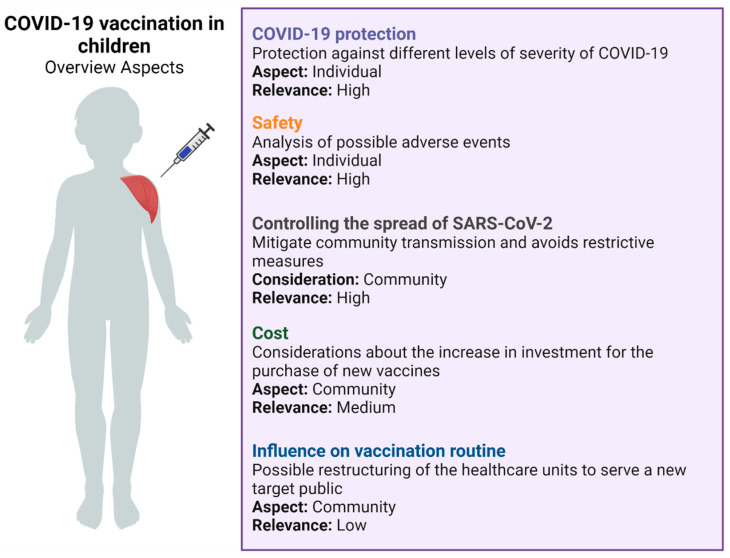
Overview about infantile vaccination for COVID-19. Adapted from Zimmerman et al. [203]. Created with BioRender.com (accessed on 5 March 2022).

It is important to highlight that, in addition to the individual long-term health consequences, the decision to vaccinate pediatric groups involves a thorough evaluation of factors such as population-level factors. From this standpoint, it is not possible to mitigate and control pandemics without the immunization of children and adolescents, since this measure also helps to mitigate community transmission, avoid restrictive measures, and support the return of pre-pandemic activities [203,204]. Besides the safety issues mentioned, there are many criteria that must be analyzed when evaluating vaccination in children. These criteria are of high, medium, or low relevance, and should be measured according to their importance as well as the reality of that population, since they are individual and community criteria (Figure 2). However, in the actual scenario, we still need more studies to confirm the long-term safety and efficacy of these vaccines in this population.

## 5. COVID-19 Future Perspectives and the Role of Vaccination in this Control

According to epidemiologists, the change in state from pandemic to endemic means that the virus will remain among the population in a reliably predictable way. In this scenario, COVID-19-related transmission, hospitalization, and death will stay stable, following a pattern. This does not necessarily mean that the number of deaths, transmissions, or even severity will get lower; just foreseeable [205]. Currently, following the advent of vaccines and the emergence of less-lethal variants, mortality rates have decreased globally. According to real-time monitoring data provided by the Center for Systems Science and Engineering (CSSE) at Johns Hopkins University, the fatality rates in February 2021 were 3.33% (466,000 daily cases and 15.4 thousand daily deaths). One year later, in February 2022, cases almost quadrupled, but the number of daily deaths declined, resulting in a death rate of 0.38% (3.18 million cases and 12,294,000 deaths) [206,207].Considering this optimistic scenario, some European countries have gradually started to adopt more flexible measures against the disease. These measures assume living with the disease, treating it as an endemic disease. In the context of epidemiological control, managing COVID-19 as an endemic disease entails not following up on all cases. In addition, countries could integrate coronavirus monitoring as with other flu-like diseases, testing representative samples, instead of every symptomatic patient [208]. However, to classify an infectious disease as endemic, the rate of infections needs to stabilize sufficiently for infection peaks (with seasonal epidemic peaks) to become predictable [209].

Therefore, although it is recognized that at some point the disease may be redefined as endemic, the WHO is currently against this position [210]. According to the organization, SARS-CoV-2 still presents many uncertainties due to its high mutation rate, and cannot yet be treated as an endemic disease. Among the key factors that remain uncertain or unknown are the duration of immunity to SARS-CoV-2 from vaccination or prior infection; whether SARS-CoV-2 will become a seasonal infection; or whether new more transmissible, immune-evading, or virulent variants will arise [209].

Currently, there is no consensus among state governments and health organizations regarding the transition to endemicity. However, most authors argue that the crucial factor for this change is the number of immunized individuals in a population [208,209]. The acquisition of effective vaccines, the intense search for antiviral drugs alternatives, and health-surveillance measures have resulted in a reduction in severe cases and fatalities. In parallel, researchers discuss the impact of the COVID-19 variants on natural immunization [211]. Telenti et al. [209] demonstrated that immune protection can affect SARS-CoV-2 transmission. Therefore, it is essential for public health-management strategies (booster doses application, incorporation of new vaccines), as well as providing a transition from pandemic to epidemic. 

In this context, wealthy countries with prominent levels of population immunity have shown lower fatality rates, leading them to consider the disease as being manageable and endemic. Remarkably, countries with different vaccinations rates per 100 inhabitants show alarming discrepancies in cumulative death rates. For comparison purposes only, high- to middle-income countries (classification as gross national income (GNI) per capita of World Bank [212]) such as Trinidad and Tobago (47th GNI position, 1.4 M inhabitants; vaccination per 100 inhabitants: 110.13; death rate: 2.72%), the Bahamas (31st GNI position, 396.91k inhabitants; vaccination per 100 inhabitants: 84.53; death rate: 2.37%), Romania (56th GNI position, 19.13 M inhabitants, vaccination per 100 inhabitants: 87.53; death rate: 2.28%); and Russia (60th GNI position, 145.9 M inhabitants, vaccination per 100 inhabitants: 112.14; death rate: 2.06%) have death rates ~ 2.8 times higher than similar-income countries presenting higher vaccination rates, such as Greece (42nd GNI position, 10.37 M inhabitants; vaccination per 100 inhabitants: 198.34; death rate: 0.9%); Italy (27th GNI position, 60.37 M inhabitants; vaccination per 100 inhabitants: 225.32; death rate: 1.08%); Portugal (37th GNI position, 10.17 M inhabitants; vaccination per 100 inhabitants: 225.45; death rate: 0.6%); Uruguay (46th GNI position, 3.49 M inhabitants; vaccination per 100 inhabitants: 231.5; death rate: 0.81%) [213].

Unfortunately, the same pattern can be seen comparing low-income countries. Countries with lower access to vaccination have shown markedly higher death rates. For comparison purposes only, Sudan (178th GNI position, 44.91M inhabitants; vaccination per 100 inhabitants: 13.65; death rate: 7.92%); Syria (160th GNI position, 18.28 M inhabitants; vaccination per 100 inhabitants: 18.44; death rate: 5.64%); and Chad (179th GNI position, 16.91 M inhabitants; vaccination per 100 inhabitants: 2.48; death rate: 2.61%) have presented death rates approximately five times higher than Rwanda (174th GNI position, 13.28 M inhabitants; vaccination per 100 inhabitants: 146.28; death rate: 1.13%); Guinea (168th GNI position, 13.5 M inhabitants; vaccination per 100 inhabitants: 44.1; death rate: 1.21%); and Togo (169th GNI position, 8.48M inhabitants; vaccination per 100 inhabitants: 32; death rate: 0.74%) [213].

However, important aspects of COVID-19 remain unknown or under investigation. These include the varying immunization rates, antibody titers’ waning rates after vaccination or natural infection, high RNA viral instability caused by mutations [214,215], and the impact of heterogeneous pandemic control in countries at distinct stages of economic development. For these reasons, predicting the transition time of COVID-19 to an endemic state remains a considerable challenge. 

It is impossible to know exactly what SARS-CoV-2 will become, but among the possible scenarios, the authors consider that most COVID-19 will turn into an influenza-like endemic disease. However, according to the WHO, annually there are 290,000 to 650,000 influenza-related respiratory deaths [216]. Given that after two years of the pandemic, the number of deaths from COVID-19 is 5.82 million [217], changing the state from pandemic to endemic will result in a better situation, but not really an ideal one. On the other hand, a more optimistic, but less probable, scenario would be SARS-CoV-2 becoming just another coronavirus species. Commonly, coronaviruses such as SARS-CoV, MERS-CoV, HCoV-NL63, HCoV-229E, HCoV-OC43, and HKU1 present a lower human health impact, with symptoms relating to mild or light respiratory illnesses [218,219]. 

Finally, the need for the equitable distribution of vaccines in different parts of the world is also highlighted. While high-income countries have a sufficient immunization rate for restrictive measures to be relaxed, low- and middle-income countries face limited and delayed access to COVID-19 vaccines, despite the existence of initiatives to this end, such as COVID-19 Vaccines Global Access (COVAX) [220]. One of the main consequences of this context is that with high infection rates around the world, coupled with the absence of broad access to vaccines and the establishment of effective immunization campaigns in certain locations, more variants of SARS-CoV-2 will continue to emerge, representing a major concern [221]. Thus, it is critical that vaccine platforms that have a rapid manufacturing process, as well as distribution without major logistical barriers and easy administration, be made widely available in order to control the advance of COVID-19 [222]. 

## 6. Conclusions 

Despite increasing advances in knowledge regarding SARS-CoV-2 and COVID-19, mass vaccination has not yet been enough to stop the pandemic. The emergence of new variants of concern remains an immense challenge, since, despite proven efficacy, vaccines for COVID-19 are not able to prevent viral infection 100% of the time. Thus, health authorities and agencies have adopted the practice of booster doses, thinking about the protection of individuals who are part of at-risk groups. Another concern about COVID-19 vaccination has been the safety of the vaccines due to their rapid development and the lack of knowledge about their long-term effects. Importantly, despite the risks attached to the available vaccines, serious adverse events such as myocarditis and pericarditis seen with RNA vaccines are considered rare, and vaccination is still recommended in the population. It has been shown that vaccination is able to prevent hospitalization of infected individuals and even reduce cases of death, and so it remains the best alternative for the pandemic to finally be considered endemic.

## Figures and Tables

**Table 1:** The characteristics of the main SARS-CoV-2 variants of concern or interest, according to WHO [27].

WHO Name	Pangos Lineage	Country of First Identification (Date)	Next Strain Clade	Number of S-Protein Mutations	Type of Variant	Concern or Characteristics
Alpha	B.1.1.7	UK (September 2020)	20I/501Y.V1	13	VOC	Rapid transmissibility and higher infectivity.
Beta	B.1.351	South Africa (May 2020)	20H/501.V2	13	VOC	Higher viral transmissibility and severity, in addition to immune escape (possible reduction in vaccine effectiveness).
Gamma	P.1	Brazil (November 2020)	20J/501Y.V3	12	VOC	Increase in viral transmissibility and possible immune escape (possible reduction in vaccine effectiveness).
Delta	B.1.617.2	India (October 2020)	21A/S:478K	15	VOC	High transmissibility and severity, in addition to a reduction in vaccine effectiveness.
Omicron	B.1.1.529	South Africa (November 2021)	21K, 21L, 21M	30	VOC	Increased viral replication, immune escape (possible reduction in vaccine effectiveness), infectivity (transmissibility), and re-infection.
Lambda	C.37	Peru (December 2020)	21G	8	VOI	Possible enhanced infectivity and immune resistance.
Mu	B.1.621	Colombia (January 2021)	21H	9	VOI	Increased transmissibility and possible immune resistance.

S—spike; UK—United Kingdom; VOC—variant of concern; VOI—variant of interest.

**Table 2:** Efficiency and neutralization activity of major COVID-19 vaccines against variants of concern after primary vaccination.

**Vaccine Name** **(Brand Name/** **Developer)**	**Type of Vaccine**		**Variants of Concern**					**References**
			**Alpha (B.1.1.7)**	**Beta (B.1.351)**	**Gamma (P.1)**	**Delta (B.1.617.2)**	**Omicron (B.1.1.529)**	
BNT162b2 (Comirnaty/Pfizer and BioNTech)	mRNA	Effectiveness (%)	93.7	74.7	75.5 *	88.0	70.0	[55,56,57,58]
		Neutralization activity (Compared to the wild-type or prototypical D614G variant **)	Practically unchanged	About 10.3-fold lower	About 3.8-fold lower	About 5.8-fold lower	About 22.0-fold lower	[59,60,61]
mRNA-1273 (Spikevax/Moderna)	mRNA		100	96.4	75.5 *	84.8	30.4	[58,62,63,64]
			Practically unchanged	About 12.4-fold lower	About 4.8-fold lower	About 8.4-fold lower	About 22.0-fold lower	[59,60,65,66]
CoronaVac (CoronaVac/Sinovac)	Inactivated Virus		Unknown	65.9	36.8	59.0	Unknown	[67,68,69]
			About 1.62-fold lower	About 3.3-fold lower	About 3.92-fold lower	About 2.34-fold lower	Neutralizing antibody titers were not found	[70,71,72,73]
BBIBP-CorV (BBIBP-CorV/Beijing Institute of Biological Products and Sinopharm)	Inactivated Virus		Unknown	Unknown	Unknown	66.9	Unknow	[74]
			About 1.4-fold higher	About 1.5-fold lower	About 1.9-fold lower	Unknown	About 10.9-fold lower	[13,73,75]
AZD-1222 (Covishield and Vaxzevria/Oxford University and AstraZeneca)	Chimpanzee adenoviral vector		74.5	21.9	64.0	67.0	71.4	[55,76,77,78]
			About 2.4-fold lower	About 9.05-fold lower	About 2.9-fold lower	About 2.5-fold lower	About 13.3-fold lower	[79,80,81]
Ad26.COV2-S (Janssen COVID-19 Vaccine/Janssen)	Human adenoviral vector		70.0	58.0	68.0	60.0	Unknown	[10,44]
			About 1.25-fold lower	About 2.97-fold lower	About 1.45-fold lower	About 1.72-fold lower	Neutralizing antibody titers were not found	[82,83]

* Estimated overall effectiveness based on the two mRNA vaccines; ** it has been shown that mutation of D614G does not significantly alter the neutralizing properties of antibodies against SARS-CoV-2, which makes vaccines developed with the wild type efficient. Therefore, different studies have compared the neutralizing activity against protein S with this substitution.

## Data Availability

Not applicable.
